# Using NGS to Uncover the Corruption of a Peptide Phage Display Selection

**DOI:** 10.3390/cimb46090627

**Published:** 2024-09-21

**Authors:** Danna Kamstrup Sell, Babak Bakhshinejad, Anders Wilgaard Sinkjaer, Ida Melissa Dawoodi, Mette Neiegaard Wiinholt, Ane Beth Sloth, Camilla Stavnsbjerg, Andreas Kjaer

**Affiliations:** 1 Department of Clinical Physiology and Nuclear Medicine, Copenhagen University Hospital-Rigshospitalet, 2100 Copenhagen, Denmark; danna@sund.ku.dk (D.K.S.); babak.bakhshinejad@sund.ku.dk (B.B.); anderssinkjaer@sund.ku.dk (A.W.S.); idamd@sund.ku.dk (I.M.D.); mette.wiinholt@sund.ku.dk (M.N.W.); anebeth@sund.ku.dk (A.B.S.); cstavnsbjerg@sund.ku.dk (C.S.); 2 Cluster for Molecular Imaging, Copenhagen University Hospital-Rigshospitalet & Department of Biomedical Sciences, University of Copenhagen, 2100 Copenhagen, Denmark

**Keywords:** biopanning, combinatorial diversity, corruption, fast-propagating clone, NGS, peptide library, phage display, sanger sequencing, sequence space, TUP

## Abstract

Phage display has been widely used to identify peptides binding to a variety of biological targets. In the current work, we planned to select novel peptides targeting CD4 through screening of a commercial phage display library (New England Biolabs Ph.D.^TM^-7). After three rounds of biopanning, 57 phage clones were Sanger-sequenced. These clones represented 30 unique peptide sequences, which were subjected to phage ELISA, resulting in the identification of two potential target binders. Following peptide synthesis, downstream characterization was conducted using fluorescence plate-based assay, flow cytometry, SPR, and confocal microscopy. The results revealed that neither of the peptides identified in the Sanger-based phage display selection exhibited specific binding toward CD4. The naïve library and the phage pool recovered from the third round of biopanning were then subjected to next-generation sequencing (NGS). The results of NGS indicated corruption of the selection output by a phage already known as a fast-propagating clone whose target-unrelated enrichment can shed light on the misidentification of target-binding peptides through phage display. This work provides an in-depth insight into some of the challenges encountered in peptide phage display selection. Furthermore, our data highlight that NGS, by exploring a broader sequence space and providing a more precise picture of the composition of biopanning output, can be used to refine the selection protocol and avoid misleading the process of ligand identification. We hope that these findings can describe some of the complexities of phage display selection and offer help to fellow researchers who have faced similar situations.

## 1. Introduction

Phage display is a widely used molecular evolution tool for the identification of peptides with binding affinity to various targets of interest [1,2,3,4]. An important milestone in the history of phage display was the construction of large libraries by using recombinant DNA technology [5,6]. This achievement was obtained by the generation of combinatorial diversity in DNA sequences [7], which turned phage display into a high-throughput selection strategy. A peptide phage display library comprises a diverse pool of phage clones that display random peptides genetically fused with one of the phage surface proteins. A pivotal concept in phage display is the physical linkage between phenotype (displayed peptide) and genotype (DNA sequence encoding the displayed peptide) [8,9,10,11,12,13]. The development and commercialization of these libraries have facilitated their widespread use across many research fields [10,11,12,13,14,15,16,17]. The affinity selection process of phage display libraries, known as biopanning, includes introducing the library to a target, washing to remove the unbound phages, elution of the bound phages, and subsequently amplification of the recovered phages through bacterial infection. Through this iterative process, peptides with target affinity are enriched and finally isolated [5,18]. To decipher the identity of isolated peptides, part of the phage genome encoding the displayed peptide is sequenced. After a few rounds of selection, the enrichment of target binders is examined by randomly picking a small number of phage clones for sequencing. Once the amino acid sequences of displayed ligands are identified, the peptides can be synthesized for further downstream characterization to verify their binding toward the target.

Although phage display selection has shown promise in the identification of many target-binding ligands, the undesirable isolation of target-unrelated peptides (TUPs) remains a major limitation of peptide discovery through biopanning. TUPs are peptides without affinity to the target of interest but are isolated because of binding to the components of the selection system (selection-related TUPs; Sr-TUPs) or due to a propagation advantage (propagation-related TUPs; Pr-TUPs) [19,20,21]. The extent of this limitation is, however, difficult to assess, as there is a scarcity of information in the literature deeply addressing the impact of TUPs on phage display selections, which can potentially lead to misleading biopanning results and interpretations. Sanger sequencing, which is economically reasonable and broadly available, has traditionally been used for the investigation of phage display selection outputs, and the scientific literature still abounds with reports on the isolation of target-binding peptides using Sanger-based phage display platforms. However, a rising interest is focusing on the utilization of next-generation sequencing (NGS) for analysis of the results of phage display selections ([22,23,24,25,26]). NGS enables the sequencing of thousands to millions of recovered clones from biopanning and allows for the investigation of a larger sequence space of the selection output. In this study, we have followed the standard methods for the identification of peptide ligands. Biopanning was carried out against immobilized recombinant human CD4 protein (hCD4), which is an interesting diagnostic and therapeutic target across a broad spectrum of diseases such as cancer, infection, and autoimmunity [27,28,29,30]. We used the Sanger approach for sequence identification and phage ELISA to identify candidate peptides for synthesis. Downstream characterization was carried out with fluorescence plate-based assay, flow cytometry, confocal microscopy, and surface plasmon resonance (SPR), but we observed no binding affinity toward the target. We subsequently sequenced the phage pool with NGS to shed light on the probable reasons for the failure to identify true target binders. The NGS analysis revealed corruption of the selection output by a fast-propagating phage clone. Our findings highlight that NGS, by providing a more complete picture of the peptide composition of biopanning output, can help in the identification of nonspecifically enriched clones that lead to the corruption of phage display selection, thus avoiding the misidentification of target-binding peptides.

## 2. Materials and Methods

### 2.1. Phage Display Library and Biopanning against Recombinant Human CD4

The Ph.D.^TM^-7 phage display peptide library (Lot number: 10043452) was purchased from New England BioLabs (Ipswich, MA, USA). This library is constructed using the M13KE phage vector and consists of random heptapeptides followed by a spacer sequence (Gly-Gly-Gly-Ser) fused to the N-terminus of the minor coat protein (pIII). The reported complexity of this library is 10^9^ sequences. Three rounds (R1–R3) of biopanning were performed using recombinant human CD4 (hCD4) protein (Sino Biological, Beijing, China) in 0.1 M NaHCO_3_ (pH 8.6) coated on a Nunc MAXIsorp 96-well plate (Thermo Fisher Scientific, Waltham, MA, USA). The recombinant hCD4 used in our work was the extracellular domain of the human CD4 consisting of 376 amino acids (with Lys 26 as the predicted N-terminal amino acid) expressed in the human embryonic kidney 293 (HEK293) cells. The protein was biotinylated and had a polyhistidine tag at the C-terminus. The quantity of target protein was reduced for stringency from 5 µg/well in round one (R1) to 2.5 µg/well in round two (R2) and 1.25 µg/well in round three (R3). Wells were blocked with 0.5% BSA for 4 h and washed six times with TBST (Tris-Buffered Saline with 0.1% Tween-20) before adding 10 µL of phage display peptide library (NEB-reported titer; 2 × 10^13^ pfu/mL) in R1, 200 µL of amplified eluate in R2, and 250 µL of amplified eluate in R3. Stringency was enhanced by increasing the number of washes and Tween-20 concentration from 10 times with 0.1% Tween in R1, to 15 times with 0.25% Tween in R2, and 20 times with 0.5% Tween in R3. Bound phages were eluted with 100 µL of 0.2 M glycine-HCl (pH = 2.2) and gentle rotation for 10 min. The phage eluate was neutralized with 15 µL of 1 M Tris-HCl (pH = 9.1) before amplification in *Escherichia coli* ER2738 culture. Non-amplified and amplified eluates were titered using the plaque count method on Tet/X-gal/IPTG LB agar plates.

### 2.2. Isolation of Phage Clones and Sanger Sequencing

Following the third round of biopanning, the amplified eluate was plated, and 57 individual well-isolated plaques were selected for sequencing. Whole phages were used as the template in PCR as described previously [31]. The PCR product was purified using the QIAquick PCR purification kit (Qiagen, Hilden, Germany) and sequenced by an ABI sequencer (Eurofins Genomics, Ebersberg, Germany).

### 2.3. Monoclonal Phage ELISA and Selection of Peptides for Synthesis

Clones were separately amplified in *E. coli* ER2738 culture (4.5 h, 37 °C, 250 rpm). After centrifugation (10 min, 25 °C, 4000× *g*) to remove bacterial cells and debris, the phage-containing supernatant was purified by adding 1/6 volume of PEG/NaCl buffer (20% PEG/2.5 M NaCl). After overnight incubation (4 °C), precipitated phages were retrieved by centrifugation (10 min, 37 °C, 12,000× *g*) and resuspended in Tris-Buffered Saline (TBS). Monoclonal phage ELISA was used to investigate the binding of 30 individual phage clones. The procedure was performed in triplicate and has been described previously [31]. Briefly, pre-blocked phages (10^10^ pfu/well) were introduced to hCD4-coated (0.5 µg/well) and non-target (PBS) wells and incubated for 0.5 h with continuous shaking. Unbound phages were removed by washing, and the bound phages were detected using an HRP-conjugated anti-M13 antibody (Sino Biological, Beijing, China) and 3,3′,5,5′-tetramethylbenzidine (Merck, Darmstadt, Germany). Absorbances at 450 (target) and 540 nm (reference) were measured in target-coated and non-target wells using a FLUOstar^®^ Omega reader (BMG Labtech, Ortenberg, Germany). A phage clone was considered a binder if the reference subtracted absorbance exceeded 0.2 in the hCD4-coated well.

### 2.4. Peptide Synthesis

Based on the monoclonal ELISA data, two peptides were selected and prepared using standard solid-phase peptide synthesis (TAG Copenhagen, Frederiksberg, Denmark). SWTVWRS (P16 M.W. = 1423.55 g/mol) and WHWPLTV (P18, M.W. = 1440.62 g/mol) peptides were synthesized with an FITC label and β-Ala or 6-aminohexanoic (Ahx) linker at the N-terminus and amidated C-terminus. Peptides were purified via high-performance liquid chromatography (HPLC, >95% purity) by TAG Copenhagen.

### 2.5. Cell Culture and CRISPR-Cas9-Mediated Construction of CD4-Knockout Cells

The human T lymphoblast cell line SUP-T1 [VB] (ATCC CRL-1942) was cultured in RPMI -1640 (#30-2001, ATCC, Manassas, VA, USA) supplemented with 10% Fetal Bovine Serum (Thermo Fisher Scientific) and 1% Penicillin–Streptomycin (Thermo Fisher Scientific). Cells were maintained at 37 °C with 5% CO_2_ at a density of 2 × 10^5^ to 2 × 10^6^ cells/mL with medium renewal every 2–3 days. Cell harvest was carried out by centrifugation of cells at 300 g for 5 min. The CRISPR-Cas9 knockout was made with the Gene Knockout Kit v2 (Synthego, Redwood City, CA, USA). Nucleofection with the SF Cell Line 4D-Nucleofector™ X Kit S (Lonza, Basel, Switzerland) was applied to deliver three single guide RNAs (sgRNAs) (AGUGCAAUGUAGGAGUCCAA, CUGGAGCUCCAGCUGAGACA, UUUUGAACUC-CACCUUCUUC) targeting exon 4 according to the manufacturer’s guidelines. Briefly, 4 µL of Supplement 1, 14 µL of SF Cell Line Nucleofector solution, 5 µg of sgRNA (30 pmol/µL), and 1 µL of Cas9 (20 pmol/µL) were incubated for 10 min to form a ribonucleoprotein (RNP) complex. For each reaction, 5 µL of cell suspension was mixed with 25 µL of RNP complex and transferred to a Nucleocuvette™ 8-well strip. Pulse code CM-198 on the 4D-Nucleofector^®^ X-unit was used to nucleofect the cells. Cells were cultured for three days before validation of knockout. DNA was extracted with the QuickExtract DNA extraction solution (Nordic Biolabs, Täby, Sweden) according to the manufacturer’s instructions. A PCR amplifying the part of the gene surrounding the cut site was carried out by mixing 25 µL of Q5^®^ High-Fidelity DNA Polymerase (NEB), 2.5 µL of 10 µM forward primer (5′-GGTTTCCT GTTGTCTGCCCT-3′), 2.5 µL of 10 µM reverse primer (5′-AGACAGGCCTCAGCTCTAGG-3′), and 200 ng of DNA in a total reaction volume of 50 µL. The following conditions were used for the reaction: initial denaturation at 98 °C for 30 s, 30 cycles of 98 °C for 10 s, 66 °C for 30 s, and 72 °C for 30 s. The PCR product was purified with the GeneJet PCR purification kit (Thermo Fisher Scientific) and sent for Sanger sequencing using Macrogen (Amsterdam, the Netherlands). Knockout success was investigated with the ICE tool (Synthego) [32] by comparing the DNA sequence from wild-type (WT) and CRISPR-Cas9-transfected cells in the targeted region. Protein knockout of CD4 was confirmed by Western blot and flow cytometry (see Supplementary Materials; Figures S1 and S2).

### 2.6. Downstream Tests

#### 2.6.1. Fluorescence Plate-Based Assay

Cells (1.5 × 10^5^ SUP-T1 WT and SUP-T1 KO) were blocked with 3% BSA in PBS (1 h, 4 °C) in wells of a U-bottom 96-well plate. Peptide solutions of 10, 20, 40, 80, 100, and 120 µM (in PBS with 1% protease-free BSA) were added, followed by 1 h incubation at 4 °C. After centrifugation (3 min, 4 °C, 600× *g*), the cells were washed twice with PBS and resuspended in 100 µL of PBS. The plate was read using a FLUOstar^®^ Omega reader with gain setting 1950.

#### 2.6.2. Flow Cytometry

SUP-T1 WT and KO cells were centrifuged at 300× *g* for 5 min and resuspended in FACS buffer (PBS with 1% BSA, 0.1% NaN_3_, 5 mM EDTA). Cells were incubated with FITC-labeled peptides (10, 20, 40, 80 µM) and 1:333 fixable viability dye eFluor 780 (Thermo Fisher Scientific) or 1:20 AF647-labeled anti-human CD4 antibody (clone OKT4, Cat# 566681, BD Biosciences, Franklin Lakes, NJ, USA) and 1:400 fixable viability dye eFluor 780 for 1 h at 4 °C. After washing three times, cells were analyzed using an LSRFortessa™ X-20 (BD Biosciences). Data analysis was performed using FlowJo^®^ software v10.4.2 (BD Biosciences).

#### 2.6.3. Confocal Microscopy

SUP-T1 WT and KO cells were transferred to a round-bottom 96-well plate at a density of 25 × 10^4^ cells per well in a complete medium. Cells were stained with Hoechst 34580 (1:2000, Invitrogen, Waltham, MA, USA), Invitrogen CellMask™ Deep Red Plasma Membrane Stain (1:1330, Thermo Fisher Scientific), and FITC-labeled peptides (20 µM) or anti-human CD4 antibody (1:10, Cat# 555346, BD Biosciences). The cells were incubated for 1 h at 4 °C in the dark, washed twice, and resuspended in Invitrogen Live Cell Imaging Solution (Thermo Fisher Scientific). Confocal imaging was performed using an LSM980 microscope (C-Apochromat objective. 40× Mag./1.2 NA, Zeiss, Oberkochen, Germany) at room temperature, and cell suspensions were transferred to an 18-well Ibidi slide (Ibidi, Gräfelfing, Germany) for imaging. Data acquisition and image analysis were carried out using Zeiss Zen Blue 3 and Zeiss Zen Black 3.7 software, respectively.

#### 2.6.4. SPR Binding Assay

The interaction between the peptides and hCD4 was investigated using a Biacore T200™ instrument (Cytiva, Marlborough, MA, USA) at Beactica Therapeutics AB (Uppsala, Sweden). hCD4 (Sino Biological) was immobilized to a CM7 chip surface (20–27 kRU) by amine coupling. Unlabeled peptides were diluted in a two-fold dilution series from 100 µM in running buffer (10 mM HEPES with pH 7.5, 150 mM NaCl, 0.05% Tween 20) with or without 1% DMSO depending on the peptide solubility. Interaction experiments were carried out at 15 °C and a flow rate of 30 μL/min. Sensorgrams were double-referenced (reference surface, blanks) before global analysis using a 1:1 interaction model including a linear drift parameter. Subsequently, peptide binding toward a reference protein (same conditions as hCD4) was used to evaluate the specificity of the peptides.

### 2.7. NGS Sample Preparation and Analysis

The single-stranded DNA of the virions from the naive library and recovered phages from the third round of selection was extracted using the NucleoSpin^®^ Plasmid kit for isolation of M13 DNA (Macherey-Nagel, Düren, Germany) and used as the template in a PCR reaction with primers and cycling conditions described previously [31]. The PCR product was purified with the QIAquick PCR purification kit (Qiagen, Germany) and underwent NGS analysis at the Center for Genomic Medicine, Copenhagen University Hospital, Rigshospitalet (Copenhagen, Denmark) using the MiSeq V3 platform. The analysis of Illumina NGS data was performed as described previously [31]. Briefly, a MATLAB script was used for read-filtering, nucleotide-to-amino acid translation, and frequency calculation. The script removed reads with invalid amino acids (containing ‘*’ or ‘X’) or incomplete linker sequences (GGGS), resulting in the division of reads into ‘cleaned’ or ‘removed’ ones.

A Python script (Supplementary Materials; Table S1) was developed to identify sequences with one amino acid difference from the WSLGYTG sequence. In short, this was achieved by identifying sequences where six out of seven amino acids matched the specific amino acid and position of the reference sequence (WSLGYTG), while the seventh amino acid was not allowed to match. This was carried out for all positions and all sequences. The identified sequences were removed from the analysis. The relative abundances were calculated through dividing the copy number of each sequence by the total number of sequences in each sample. This was performed for both the naïve library and the eluate sample.

## 3. Results

### 3.1. Selection of Phage Display Library against hCD4

To select hCD4-binding peptides, three rounds of biopanning were carried out using the Ph.D.^TM^-7 phage display library. To determine the phage recovery efficiency, the phage pool obtained from each round of biopanning was titered (Table 1). The recovery efficiency was calculated via dividing the titer of the output phage (eluate from each round) by the titer of the input phage. Our results indicated an increase in the recovery efficiency after each round from 1.6 × 10^−6^ in round one to 3.4 × 10^−4^ in round three, suggesting an enrichment of the phage population during rounds of selection.

The eluate from the third round of selection was plated and 57 randomly picked phage clones producing blue plaques were sequenced, representing 30 unique sequences (Table 2).

### 3.2. Screening for Target Binding of Isolated Phage Clones by Phage ELISA

**Figure 1:**
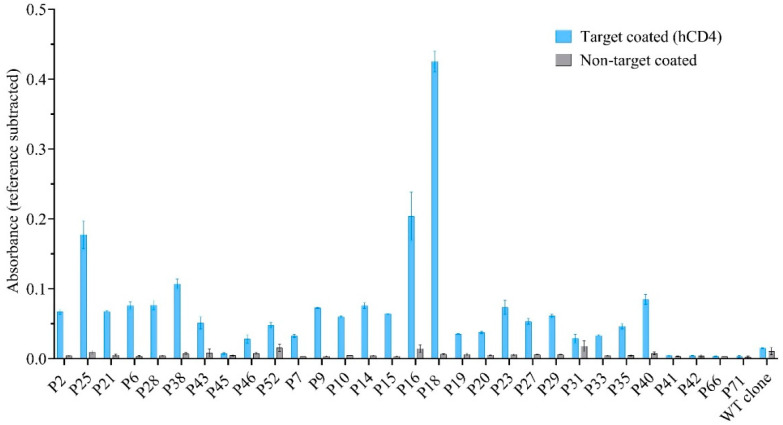
Phage ELISA for screening of target binding of phage clones (listed based on frequency in biopanning from left to right). Target-coated wells were incubated with phages representing each unique peptide sequence or a WT clone (without displayed peptide). Clones displaying the peptide sequences SWTVWRS (P16) and WHWPLTV (P18) indicated reference subtracted absorbances above 0.2 and were considered potential target binders (n = 3).

To evaluate the binding of the isolated phage clones, 30 phage clones (covering every unique sequence identified) were amplified and investigated by phage ELISA. The phage ELISA showed a higher signal in the hCD4-coated wells for the phage clones displaying the peptide sequences P16 and P18 compared to the other clones (Figure 1). Based on this, the two candidate sequences were synthesized for further downstream characterization to investigate whether the sequences could maintain target binding as free peptides. Since the peptides need to bind to the CD4 receptor expressed on cells, we chose a CD4-expressing cell line (as the target cell) and made a CD4 knockout cell line (as the control cell) for these assays.

### 3.3. Evaluation of Binding of Synthetic Peptides by Cell-Based Assays

**Figure 2:**
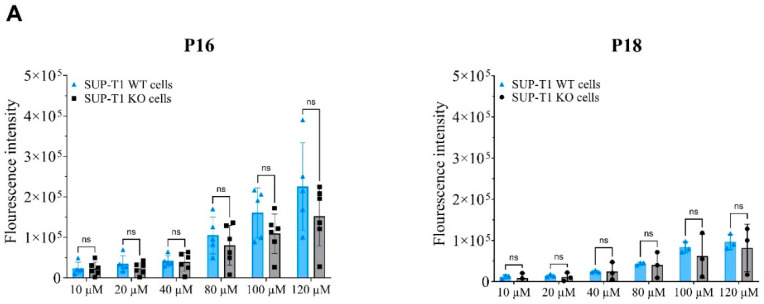

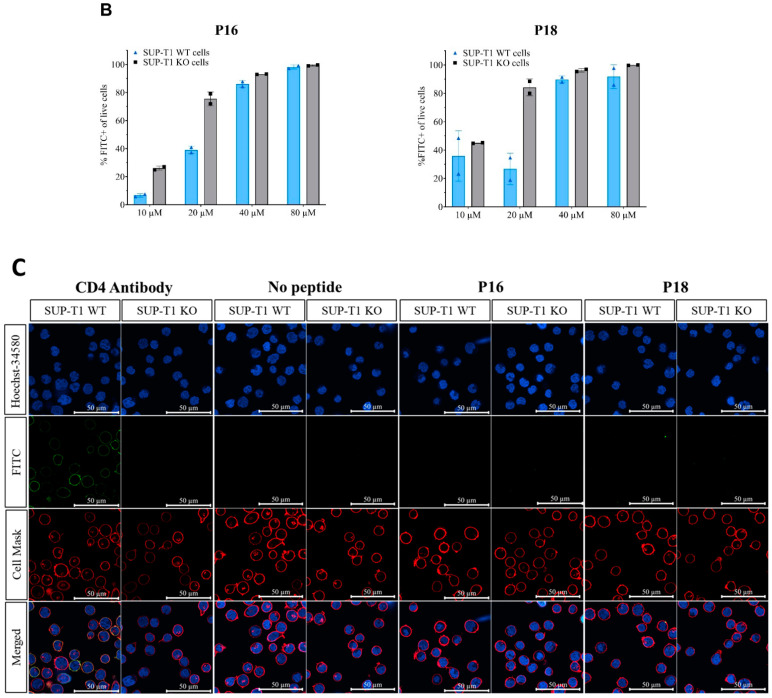
Investigation of the binding affinity of the synthesized FITC-labeled phage display-derived peptides P16 and P18 by comparison of binding between CD4-expressing (SUP-T1 WT) and -non-expressing (SUP-T1 KO) cells. ns: nonsignificant (**A**) Shows fluorescence intensity measurements in plate assay (P16: n = 5, P18: n = 3). Statistical analysis using two-way ANOVA (data tested for normal distribution) followed by Šídák’s multiple comparisons test revealed no significant group differences (*p* < 0.05). Error bars represent mean ± standard deviation (SD). (**B**) Shows flow cytometry analysis of the percentage of FITC-positive cells (% of live, n = 2). Error bars represent mean ± SD. (**C**) Shows confocal microscopy images of cells incubated with FITC-labeled peptides (P16 and P18) or anti-human CD4 antibody (green). Hoechst 34580 (blue = nuclei) and CellMask™ (red = membrane).

We made a CRISPR-Cas9 knockout of CD4 in the CD4-expressing lymphoblast cell line SUP-T1 to use as a control in the cell binding experiments (Supplementary Materials; Figures S1 and S2). The peptides P16 and P18 were FITC-labeled, and their binding to the two cell lines was measured by the increased fluorescence signal of the WT cell line compared to the CD4-knockout (KO) cell line. The binding of P16 and P18 was assessed by plate-based assay (Figure 2A), flow cytometry (Figure 2B), and confocal microscopy (Figure 2C).

The plate assay did not show a significantly higher fluorescence signal for SUP-T1 WT cells compared to SUP-T1 KO cells, although a tendency toward a higher signal can be observed for the 120 µM peptide concentration for P16 (Figure 2A). The flow cytometry assay indicated no significant difference between the binding of peptides to SUP-T1 WT and SUP-T1 KO cells. Even a tendency toward higher SUP-T1 KO cell binding was observed for both peptides (Figure 2B). Confocal imaging with a FITC-labeled anti-CD4 antibody showed fine membrane staining; however, no fluorescence signal was observed after incubation of the cells with FITC-labeled peptides (Figure 2C).

### 3.4. Evaluation of Binding of Synthetic Peptides to Recombinant CD4 Protein by SPR

**Figure 3:**
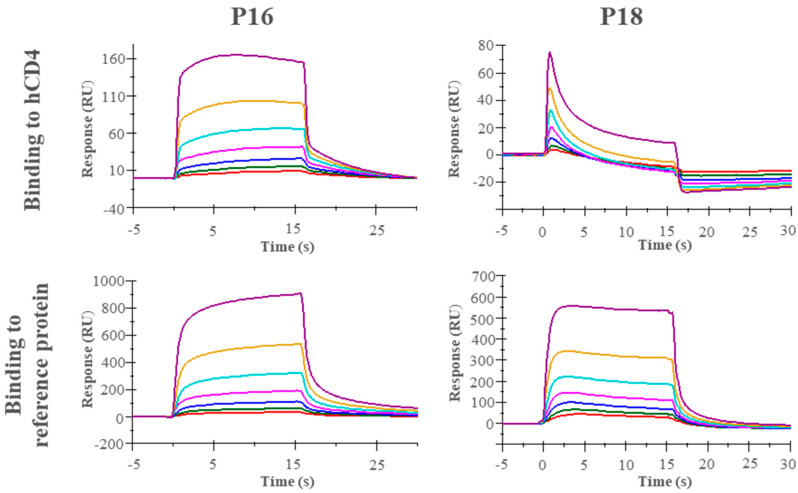
hCD4 was immobilized on a CM7 surface chip, and the peptide solutions (purple 100 µM, yellow 50 µM, turquoise 25 µM, pink 12.5 µM, blue 6.25 µM, green 3.13 µM, and red 1.56 µM) were run to test the affinity of the peptides toward hCD4 (**upper panel**) and a reference protein (**lower panel**).

The binding affinity of unlabeled peptides toward recombinant hCD4 was characterized by SPR assay. The hCD4 and reference protein were immobilized, and peptide binding events were recorded for a series of 2-fold dilutions from 100 µM peptide. Subsequently, data were plotted to generate binding curves. The results of SPR (Figure 3) showed a higher signal (response units) for the reference protein compared to hCD4, indicating nonspecific binding of peptides P16 and P18.

### 3.5. NGS Analysis of the Naïve Library and Recovered Eluate from the Third Round of Selection

As a result of the inability to identify target-binding peptides through the Sanger-based phage display methodology, NGS was conducted on the naïve Ph.D.^TM^-7 library and the third-round eluate (Table 3).

**Figure 4:**
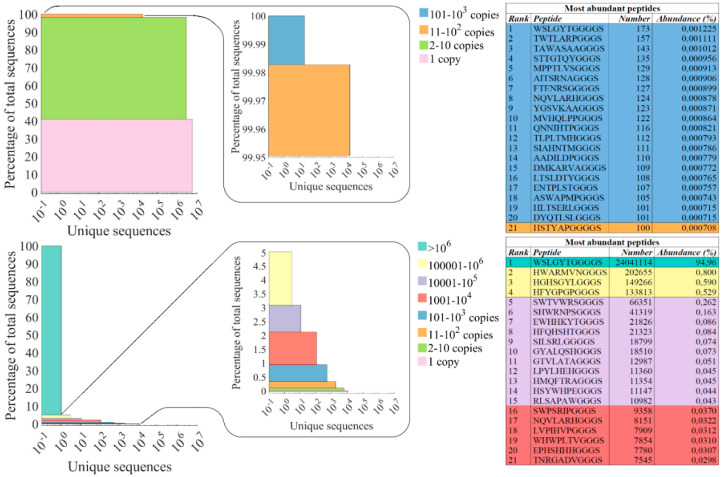
The abundance of clones in the naïve Ph.D.^TM^-7 library (**top**) and the 3rd round eluate (**bottom**) are presented as stacked bar plots. The height of each bar is proportional to the percentage of each group of peptide sequences in the phage pool, and the width of each bar corresponds to the number of unique sequences. The zoomed-in part provides a more detailed visualization of smaller bins that cannot be clearly distinguished in the stacked bar plot of the 3rd round eluate. The topmost sequences of each dataset are indicated in the tables on the right (with colors corresponding to the bins they are derived from). The NGS datasets used for the preparation of stacked bar plots are available as CSV files in the Supplementary Materials. These files contain the sequence, copy number (frequency), and relative abundance (%) of the peptide sequences.

The acquired NGS reads from the naïve Ph.D.^TM^-7 library and the third-round eluate were segregated into bins based on sequence abundance, and the resulting data are illustrated in stacked bar plots (Figure 4), inspired by the work of Matochko et al. [33].

The NGS analysis of the third-round eluate revealed a substantial corruption of the phage pool. A staggering 95% of the phage pool was represented by the clone displaying the peptide sequence WSLGYTG (the turquoise bin in the naïve library plot). Interestingly, the WSLGYTG-displaying clone was also the most abundant in the naïve Ph.D.^TM^-7 library; however, its frequency does not stand out in comparison with the remaining topmost abundant clones of the naïve Ph.D.^TM^-7 library (see table of the most abundant peptides of naïve Ph.D.^TM^-7 library in Figure 4).

A substantial decline in the diversity of the third-round eluate compared to the naïve library can be seen in the horizontal axis of the plot that represents the number of unique (distinct) sequences for different bins. Also, Table S2 (in the Supplementary Materials) indicates that the percentage of distinct sequences is largely diminished (from 61.6% to 0.08%) after three rounds of biopanning. Additionally, singletons (sequences with frequency = 1) make a larger contribution to the population of distinct sequences in the naïve library (Table S2). By comparing the percentage of singletons, the diversity of the naïve Ph.D.^TM^-7 library and the third-round eluate can be assessed to provide a more complete picture of the composition of phage pools. After three rounds of biopanning, the percentage of singletons is greatly reduced from 41% in the naïve Ph.D.^TM^-7 library to 0.04% in the third-round eluate, as indicated in Table S2 and the lowest bin (pink color) of stacked bar plots in Figure 4. This large reduction in the percentage of singletons and consequently the diversity of the eluate is also coupled with the appearance of a subpopulation of highly frequent peptides (peptides with frequency >10^3^) in the third-round eluate, which is reflected by the emergence of new bins in the stacked bar plot of this phage pool.

### 3.6. Comparison of the Frequency of Identified Peptides between Sanger Sequencing and NGS Datasets

**Figure 5:**
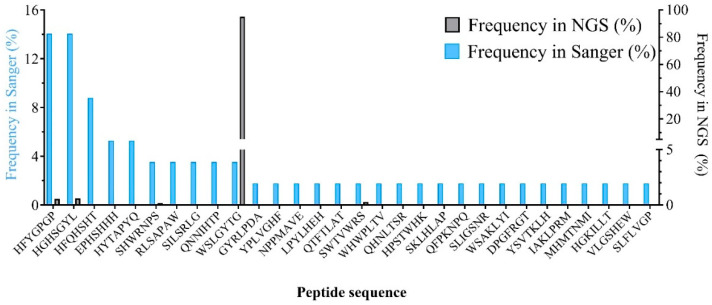
Plot showing a comparison of the frequency of identified peptides in Sanger sequencing (blue, left *y*-axis) and NGS (black, right *y*-axis) datasets.

In Figure 5, we made a comparison between the frequency of Sanger-identified peptides in Sanger and NGS datasets. Also, Table S3 (in the Supplementary Materials) contains information about the rank, absolute frequency (copy number), and relative frequency (percentage) of these peptides in the NGS dataset.

As indicated in Figure 5 and Table S3, peptides identified by plating of the eluate and Sanger sequencing of randomly chosen clones possess a wide range of frequencies and ranks in the NGS dataset. Eleven of the peptides identified by the Sanger methodology cannot be found in the top 100 sequences of NGS, and three peptides cannot be found even in the top 1000 sequences of NGS, which is evidence of the huge sampling bias of the Sanger approach. Importantly, Figure 5 illustrates the inability of Sanger sequencing to detect library corruption, even though the WSLGYTG-displaying clone constitutes around 95% of the third-round eluate.

## 4. Discussion

In the current work, we performed the traditional practice for phage display selection of peptide ligands. Following three rounds of biopanning, Sanger sequencing (list of isolated peptides in Table 2), and phage ELISA (Figure 1), two candidate peptides were synthesized and tested in downstream assays (Figure 2 and Figure 3). Downstream characterization of peptides demonstrated the lack of target-specific binding. Special attention must be paid to distinguish the genuine lack of binding from poorly designed and executed experiments when reporting non-binding outcomes. In our study, we applied a variety of binding assays covering labeled peptides interacting with cell-surface-expressed CD4 (plate assay, flow cytometry, and confocal microscopy) and unlabeled peptides interacting with recombinant protein (SPR). We chose assays that are commonly used to evaluate the binding of peptides identified by phage display. Since we did not have a positive control (i.e., a peptide that we knew bound to CD4), we validated all our cell-based assays with a known peptide, Tyr3-Octreotate, and its interaction partner, somatostatin receptor 2 (SSTR2) (Supplementary Materials; Figure S3). Since we were able to demonstrate the binding of FITC–Octreotate to SSTR2-expressing cells, we believe that the absence of CD4 binding is attributed to the minimal or nonexistent affinity of the selected peptides, rather than erroneous assays.

As phage display coupled with Sanger sequencing remains widely used for the identification of peptide ligands [34,35,36], it raises the question of whether other researchers are encountering challenges in isolating peptides with true target affinity. We developed a CD4 KO cell line to have a controlled experimental environment where peptide binding can be compared in the presence and absence of CD4. We believe that this is an ideal control to validate the specificity of binding interaction. We do recognize that such a control is not always available and therefore the most appropriate available control is used. Typically, this is a cell line that is similar to the target cell yet differs in terms of CD4 expression. However, variation in the diversity of receptor expression also exists, and thus what may seem like target specificity (no binding to the control cell line) may be explained by the differential expression of receptors.

Several factors can contribute to the isolation of peptides not exhibiting target binding affinity. Firstly, conducting a peptide binding assay on a cell-expressed protein while performing biopanning on a recombinant protein potentially introduces confounding effects. Proteins, recombinant or cell-surface-expressed, adopt different conformations in various environments. Immobilizing a recombinant protein by adsorption (as carried out in biopanning) might have altered the conformation [37], leading to discrepancies in binding behavior between biopanning and the cell-based downstream assays. Furthermore, the recombinant hCD4 used in our work was a biotinylated protein fused with a polyhistidine tag. These features might lead to some unwanted changes in the protein structure. Secondly, the isolated peptides could be nonspecific binders (lacking selectivity toward CD4), which explains the similar binding results for the SUP-T1 WT and SUP-T1 KO cells in the plate assay and flow cytometry (Figure 2A,B) and the comparable binding curves for recombinant CD4 and the reference protein in SPR (Figure 3). Thirdly, the risk of isolating target-unrelated peptides such as Sr-TUPs and Pr-TUPs remains a major limitation in phage display selections [19,20,21]. A sufficient selection pressure (stringency) is needed to effectively remove Sr-TUPs from the pool while maintaining target-specific binders. However, the balance of stringency is difficult to determine before biopanning experiments and is left to be found in a trial-and-error process. An insufficient selection pressure fails to remove the low-affinity binders, which results in the remaining phage pool consisting of both high- and low-affinity binders. This diminishes the chance of picking out high-affinity binders when selecting clones for Sanger sequencing. Nonetheless, applying an overly stringent selection could also result in the elimination of potential binders from the pool. Removal of Pr-TUPs is impossible even by the conventional way of enhancing the stringency of selection mentioned above. These clones are undesirably enriched in the selection output due to a higher propagation capacity [21].

Undetected in the Sanger sequencing results, the NGS analysis revealed a high level of selection corruption by a clone displaying the peptide WSLGYTG (Figure 4 and Figure 5). We have previously reported this clone as a fast-propagating phage whose predominance in the selection output is due to a propagation advantage associated with the genomic deletion of the lacZ gene (detected by whole-genome sequencing) and not true binding affinity to the target [31]. This finding contradicted the instructions of the library manufacturer to ascribe the appearance of white plaques to the contamination of the phage stock with environmental M13 phages during panning and amplification. Contrastingly, our analyses provided evidence for the notion that the phage producing white plaque on the ITPG-Xgal agar plates originates from the library itself. Further investigation through phage ELISA confirmed that this clone does not indicate significant binding to the target protein, and phenotypic characterization through competitive propagation assay, by inoculating approximately equal titers of the clone and a random pool of the library (representing an average propagation rate of the clones in the library) into an early log *E.coli* culture, revealed the faster propagation rate of the WSLGYTG-displaying phage compared to the library clones. Clones with a propagation advantage could mislead researchers to consider them as target binders. Their target-unrelated predominance in the pool can also outcompete the clones displaying true target binders and prevent their enrichment in the selection output. The substantial corruption of the phage pool by the WSLGYTG-displaying clone can provide a rigorous explanation for the misidentification of target-binding peptides. Furthermore, the presence of fast-propagating clones reduces the diversity of the phage library during the amplification steps between selection rounds [38]. Slow-propagating clones will thus be present in lower copy numbers and are more likely to be lost in the phage pool, despite potentially displaying target-binding peptides. The negative consequence of the presence of fast-propagating phage clones and the subsequent decreased diversity (as seen by a reduction in the percentage of the singleton population and a decrease in the number of unique sequences in Figure 4) result in the loss of many promising hits from the pool. It is worth mentioning that there is a distinction between favorable and unfavorable declines in diversity. The favorable decline in diversity arises from the removal of phages whose displayed peptides do not bind to the target. This type of diversity loss is an inherent feature of phage display selection and allows for the convergence of the peptide pool to a smaller subpopulation of target-binding sequences. However, what was observed in our work was an unfavorable decline in diversity that can cause bias in the composition of the selection output by target-independent enrichment of nonspecific binders in the phage pool. We used different sequencing depths with 14.13 × 10^6^ and 25.32 × 10^6^ reads for the naïve library and third-round eluate, respectively (Table 3). We have previously indicated that increased sequencing depth can lead to observing a lower diversity (lower percentage of singleton and distinct sequences) in the same phage pool [39]. Therefore, a portion of the diminished diversity observed in the eluate can be attributed to the higher sequencing depth of NGS used for the analysis of this sample. However, the substantial decline in the eluate diversity (shown by the reduced percentage of singletons from 41% in the naïve library to 0.04% in the eluate and the reduced percentage of distinct sequences from 61.6% in the naïve library to 0.08% in the eluate; Supplementary Materials Table S2) provides strong evidence for the notion that only a minor fraction of the observed reduced diversity in the eluate derives from discrepancies in the sequencing depth and a major portion of it results from the genuine collapse in the diversity that has happened during rounds of panning. As the WSLGYTG-displaying clone had a high frequency in the naïve library due to its enhanced propagation rate, biopanning on CD4 faced an inherent challenge during selection rounds. Additionally, once the naive library is subjected to several rounds of selection and amplification, a new subpopulation of overabundant peptides emerges in the eluate. This subpopulation contains sequences with a high frequency (peptides with a frequency over 1000 in Figure 4). Some of these highly frequent clones likely have a high propagation capacity but less than the WSLGYTG-displaying clone. Due to not having a targetless control (the naïve library undergoing rounds of serial amplification without target exposure), we cannot decisively mention how many of these clones are genuine fast propagators whose enrichment in the eluate derives from a high propagation rate, not binding affinity to the target. This subpopulation of highly frequent peptides might also play a role, albeit to a lower degree than the WSLGYTG-displaying clone, in the corruption of the pool. The (serial) amplification of the naïve library can prove useful for prospective mapping of these fast-propagating clones, thus avoiding interfering with the identification of target-specific binders in the biopanning output.

The corruption was not detected or even suspected through Sanger sequencing (Figure 5), which is explained by the limited sequence space of the eluate covered by this approach. This finding exemplifies the key beneficial value of NGS compared to Sanger sequencing, as NGS explores a broader sequence space and provides a more precise picture of the composition of the biopanning output. Given the size of the third-round eluate (9.8 × 10^7^ pfu/mL) (Table 1) and the depth of the sequencing platform used for the analysis of this sample (25.32 × 10^6^ reads; Table 3), we can conclude that a noticeable fraction of the sequence space of the third-round eluate has been covered by our sequencing strategy. Of note, even an increased recovery efficiency (Table 1), which indicates the enrichment of the phage population during selection rounds, could not provide any clues on the pool corruption. If the third-round eluate corruption had been detected at an earlier stage, the decision to proceed with downstream analysis of the selected peptides might have been reconsidered or even abandoned. Although target-specific binders are likely to exist in the recovered phage pool, the shocking extent of the corruption raises doubts about advancing with any of the peptide sequences identified through NGS. Our results reinforce the notion of how the distorted image of the selection output portrayed by the Sanger sequencing methodology can misguide the phage display research for finding target-specific peptides.

## 5. Conclusions

Phage display has shown enormous promise in ligand identification and heralded new advances in the discovery of peptides with diagnostic and therapeutic applications. However, it is likely that challenges will be met on the ligand discovery road using phage display. Differentiating genuine target binders and false positives is a crucial step in enhancing the integrity of phage display selections. Our findings exemplify the importance of reporting not only successful phage display selections but also biopanning experiments where the isolated peptides fail to bind to the target of interest. Also, our study highlights the importance of trying to explore the reasons behind the observed failure, as it can help advance the understanding of the selection process and optimize the phage display protocols. Choosing suitable phage display libraries, using appropriate selection conditions (balanced stringency), validating downstream assays, and applying proper controls in both the selection process and downstream characterization are all determining factors that impact the quality of the peptides identified in phage display selections. More importantly, the sequencing strategy used for analyzing the results of selection and choosing candidate peptides for downstream binding characterization plays a major role in troubleshooting the selection process and improving the identification of promising hits. In this context, NGS can overcome some of the bottlenecks associated with the commonly used Sanger sequencing approach and avoid misidentification of the nonspecifically enriched TUPs. We believe that counterculture reports, like the current study, can make a significant contribution to our deeper understanding of the anatomy of the fall and rise of a phage display library selection. Beyond doubt, the constructive debates ignited by such reports that are born from less-than-optimal results can be eye-openers for phage display scientists. Based on this, we hope that our work will shine light on some of the less-discoursed aspects of phage display in peptide discovery.

## Figures and Tables

**Table 1:** The recovery efficiency of the phage pool during rounds of selection against hCD4.

Biopanning Round	Input Phage (pfu/mL)	Output Phage (pfu/mL)	Recovery Efficiency
1	2.0 × 10^13^	3.1 × 10^7^	1.6 × 10^−6^
2	2.5 × 10^10^	1.2 × 10^6^	4.8 × 10^−5^
3	2.9 × 10^11^	9.8 × 10^7^	3.4 × 10^−4^

**Table 2:** Thirty unique peptides encoded by sequenced phages and their frequency (absolute number and percentage) recovered from the 3rd round of biopanning toward hCD4.

Peptide ID	Peptide Sequence	Frequency of Occurrence	Frequency in Percentage (%)
P2	HFYGPGP	8	14.0
P25	HGHSGYL	8	14.0
P21	HFQHSHT	5	8.8
P6	EPHSHHH	3	5.3
P28	HYTAPYQ	3	5.3
P38	SHWRNPS	2	3.5
P43	RLSAPAW	2	3.5
P45	SILSRLG	2	3.5
P46	QNNIHTP	2	3.5
P52	WSLGYTG	2	3.5
P7	GYRLPDA	1	1.8
P9	YPLVGHF	1	1.8
P10	NPPMAVE	1	1.8
P14	LPYLHEH	1	1.8
P15	QTFTLAT	1	1.8
P16	SWTVWRS	1	1.8
P18	WHWPLTV	1	1.8
P19	QHNLTSR	1	1.8
P20	HPSTWHK	1	1.8
P23	SKLHLAP	1	1.8
P27	QFPKNPQ	1	1.8
P29	SLIGSNR	1	1.8
P31	WSAKLYI	1	1.8
P33	DPGFRGT	1	1.8
P35	YSVTKLH	1	1.8
P40	IAKLPRM	1	1.8
P41	MHMTNMI	1	1.8
P42	HGKILLT	1	1.8
P66	VLGSHEW	1	1.8
P71	SLFLVGP	1	1.8

**Table 3:** Overview of the NGS data showing the filtering of reads (into cleaned and removed reads) and the number of unique reads in the naïve Ph.D.^TM^-7 library and the 3rd round eluate. The cleaned reads were used for the preparation of stacked bar plots.

Sample	Total Number of Reads	Number of Cleaned Reads	Number of Removed Reads	Percentage of Removed Reads	Total Number of Unique Reads	Number of Cleaned Unique Reads	Number of Removed Unique Reads
**Naïve Ph.D.^TM^** **-7**	16,136,813	14,126,481	2,010,332	12.46%	9,343,121	8,706,438	636,683
**3rd round eluate**	26,352,345	25,317,417	1,034,928	3.93%	42,877	19,333	23,544

## Data Availability

The NGS datasets of the naïve library and the -third-round eluate are available as CSV files in the Supplementary Materials.

## Supplementary Materials

The following supporting information can be downloaded at: https://www.mdpi.com/article/10.3390/cimb46090627/s1, Table S1: Python script used to identify sequences with one amino acid difference from the WSLGYTG sequence; Table S2: The number and percentage of singleton, repeated, and distinct sequences in the naïve library and 3rd round eluate; Table S3: The rank, absolute frequency, and relative frequency of Sanger-isolated peptides in the NGS dataset; Validation of CD4-KO cells; Figure S1: Western blot analysis of CD4 KO cells; Figure S2: Flow cytometric validation of CD4 KO cells; Figure S3: Investigation of binding of FITC-Octreotate to H69 and SUP-T1 cells. Files of NGS data: The NGS datasets of the naïve library and the 3rd round eluate are available as CSV files.
