# Supplementary material for: Using NGS to Uncover the Corruption of a Peptide Phage Display Selection
Source: Curr Issues Mol Biol. 2024 Sep 21;46(9):10590–605. doi: 10.3390/cimb46090627 (PMC11431649; doi:10.3390/cimb46090627)
Supplement: Supplementary file 1 [file cimb-46-00627-s001.zip › Supplementary Materials.pdf]

Supplementary materials

Supplementary Table S1: Python script used to identify sequences with one amino acid difference from the WSLGYTG sequence.

```
# -*- coding: utf-8 -*-
"""
Created on Tue Dec  5 10:13:00 2023
@author: zmn159
"""
'''Find all WSL misreads with one AA difference but do not remove WSLGYTGGGGS true
clone'''
import os
import pandas as pd

'''Desktop location:'''
data_root = r'N:\SUN-BMI-CMI\Study database\Danna Kamstrup Sell
(DKS)\DKS_017_2308_Manu3\Results\NGS\Stacked barplot'

'''Paths'''
Sample_root = os.path.join(data_root, 'Sample_AA_Clean.csv')
Naive_root = os.path.join(data_root, 'NL_Depth_Sample_AA_Clean.csv')

'''Load data'''
row_num = 10000000
# one_df = pd.read_csv(Sample_root, nrows=row_num)
# OG_df = pd.read_csv(Sample_root, nrows=row_num)

one_df = pd.read_csv(Naive_root, nrows=row_num)
OG_df = pd.read_csv(Naive_root, nrows=row_num)
N = sum(OG_df['Number'])

'''Calculate the relative percentage column and update it'''
one_df['Relative (%)'] = (one_df['Number']/(one_df['Number'].sum()))*100
OG_df['Relative (%)'] = (OG_df['Number']/(OG_df['Number'].sum()))*100

'''Add a rank column - used for merge later'''
one_df['Rank'] = one_df['Relative (%)'].rank(ascending=False, method='first')
OG_df['Rank'] = OG_df['Relative (%)'].rank(ascending=False, method='first')


'''Find WSL misreads with one AA misreads of WSLGYTGGGGS but exclusing
WSLGYTGGGGS'''
one_df = OG_df[OG_df['AA'].str.contains('(?![W])[A-Z]SLGYTGGGGS')==True]
two_df = OG_df[OG_df['AA'].str.contains('W(?![S])[A-Z]LGYTGGGGS')==True]
three_df = OG_df[OG_df['AA'].str.contains('WS(?![L])[A-Z]GYTGGGGS')==True]
four_df = OG_df[OG_df['AA'].str.contains('WSL(?![G])[A-Z]YTGGGGS')==True]
five_df = OG_df[OG_df['AA'].str.contains('WSLG(?![Y])[A-Z]TGGGGS')==True]
```

```
six_df = OG_df[OG_df['AA'].str.contains('WSLGY(?![T])[A-Z]GGGGS')==True]
seven_df = OG_df[OG_df['AA'].str.contains('WSLGYT(?![G])[A-Z]GGGS')==True]


'''Concat all df's'''
concatted_df = pd.concat([one_df, two_df, three_df, four_df, five_df, six_df, seven_df],
axis=0)

'''Drop duplicates rank columns in the concat df'''
dropduplicates_df = concatted_df['Rank'].drop_duplicates(keep='first')

'''Merge on Rank column into originl df'''
WSL_df = pd.merge(dropduplicates_df, OG_df, on="Rank")

'''Concat OG and WSL_df df's'''
OG_WSL_concat_df = pd.concat([OG_df, WSL_df], axis=0)

'''Drop ALL duplicates rank columns between OG_df and WSL_df (OG_WSL_concat_df)'''
WSL_removed_df = OG_WSL_concat_df['Rank'].drop_duplicates(keep=False)

'''Merge on Rank column into originl df'''
WSL_removed_df = pd.merge(WSL_removed_df, OG_df, on="Rank")

'''Update the relative percentage column and update it'''
WSL_removed_df['Relative (%)'] =
(WSL_removed_df['Number']/(WSL_removed_df['Number'].sum()))*100

'''Delete Rank column'''
WSL_removed_df = WSL_removed_df.drop('Rank', axis=1)

'''Print files with WSL one AA misread clones removed from as csv files'''
# WSL_removed_df.to_csv('Removed_WSL_Sample_AA_Clean.csv', index=False)
WSL_removed_df.to_csv('Removed_WSL_NL_Depth_Sample_AA_Clean.csv', index = False)

'''Percentages of removed WSL misreads of total removed reads'''
x = sum(WSL_df.Number)
y = sum(OG_df.Number)
z = (x/y)*100
```

Supplementary Table S2. The number and percentage of singleton, repeated, and distinct sequences in the naïve library and 3rd round eluate

Sample	Percentage of singletons	Percentage of repeated* sequences	Percentage of distinct sequences
**Naïve Ph.D.-7**	40.99	20.64	61.63
**3rd round eluate**	0.042	0.033	0.075

*Included one copy of each repeated sequence and excluded the remaining copies of that sequence

Supplementary Table S3: The rank, absolute frequency, and relative frequency of Sanger-isolated peptides in the NGS dataset

Peptide ID	***Peptide sequence***	***Rank in NGS***	***Absolute frequency in NGS***	***Relative frequency in NGS***
P2	HFYGPGP	4	133813	5.29E-01
P25	HGHSGYL	3	149266	5.90E-01
P21	HFQHSHT	8	21323	8.42E-02
P6	EPHSHHH	20	7780	3.07E-02
P28	HYTAPYQ	36	3941	1.56E-02
P38	SHWRNPS	6	41319	1.63E-01
P43	RLSAPAW	15	10982	4.34E-02
P45	SILSRLG	9	18799	7.43E-02
P46	QNNIHTP	60	2525	9.97E-03
P52	WSLGYTG	1	24041114	9.50E+01
P7	GYRLPDA	180	609	2.41E-03
P9	YPLVGHF	43	3399	1.35E-02
P10	NPPMAVE	1255	33	1.30E-04
P14	LPYLHEH	12	11360	4.49E-02
P15	QTFTLAT	185	589	2.33E-03
P16	SWTVWRS	5	66351	2.62E-01
P18	WHWPLTV	19	7854	3.10E-02
P19	QHNLTSR	29	5264	2.08E-02
P20	HPSTWHK	24	6175	2.44E-02
P23	SKLHLAP	421	202	7.98E-04
P27	QFPKNPQ	Not found in NGS analysis		
P29	SLIGSNR	306	295	1.17E-03
P31	WSAKLYI	1426	27	1.07E-04
P33	DPGFRGT	67	2040	8.06E-03
P35	YSVTKLH	267	357	1.41E-03
P40	IAKLPRM	25	5971	2.36E-02

P41	MHMTNMI	244	400	1.58E-03
P42	HGKILLT	102	1322	5.22E-03
P66	VLGSHEW	1719	20	7.90E-05
P71	SLFLVGP	789	70	2.76E-04

Validation of CD4-KO cells

For the validation of CD4-KO, western blot and flow cytometry analysis were conducted (Figure S1 and S2). For western blot, SUP-T1 WT and KO cells were harvested, centrifuged at 300 g for 5 min, and resuspended in cold Pierce RIPA buffer (Thermo Fisher Scientific) with Halt Protease Inhibitor Cocktail (Thermo Fisher Scientific). Cells were transferred to a CKMix tube and homogenized with Precellys Evolution Homogenizer (Bertin Technologies, France) at 6000 rpm for 20 s at 4°C. Samples were incubated on ice for 30 min and centrifuged at 14000 g for 10 min. Protein concentration in the supernatant was determined using the Pierce™ BCA Protein Assay Kit (Thermo Fisher Scientific) following the manufacturer's protocol. The samples were denatured with Bolt LDS Sample buffer and Bolt Reducing Agent (Thermo Fisher Scientific) and loaded on a Bolt 4-12% Bis-Tris Plus gel with a SeeBlue Plus2 Prestained standard. The gel was run at 200 V for 22 min, placed on an iBlot™ 2 Transfer Stack (Thermo Fisher Scientific) containing a membrane, and placed inside the iBlot™ 2 Gel Transfer Device (Thermo Fisher Scientific). After blotting, the membrane was immersed in the iBind™ Flex Solution (Fisher Scientific). The membrane was incubated with primary rabbit anti-CD4 antibody diluted 1:200 (#D2E6M, Cell Signaling Technology, USA), mouse anti-β-actin antibody diluted 1:200 (#3700S, Cell Signaling Technology, USA), anti-rabbit HRP-conjugated secondary antibody diluted 1:500 (#7074P2, Cell Signaling Technology, USA) and an anti-mouse HRP-conjugated secondary antibody diluted 1:500 (#7076S, Cell Signaling Technology, USA) in the iBind™ Flex Western Device (Thermo Fisher Scientific) overnight at 4°C. The membrane bands were developed using the SuperSignal™ West Pico PLUS Chemiluminescent Substrate (Thermo Fisher Scientific), according to the manufacturer's instructions. Imaging of the chemiluminescent signal was performed on the ImageQuant™ LAS 500 (GE Healthcare Life Sciences, USA). For flow cytometry, SUP-T1 WT and KO cells were centrifuged at 300 g for 5 min and resuspended in FACS buffer (PBS with 1 % BSA, 0.1 % NaN_3, 5 mM EDTA). Cells were

incubated with CD4 antibodies (clone OKT4, Cat# 566681; clone RPA-T4, Cat# 561844; L200, Cat#562842; SK3, Cat#565994, BD Biosciences, USA) for 30 min at 4°C. After washing three times, cells were analyzed using an LSRFortessa™ X-20 (BD Biosciences). Data analysis was done using FlowJo® software v10.4.2 (BD Biosciences). Western blot and flow cytometric analysis show that CD4 knock out was achieved at protein levels.

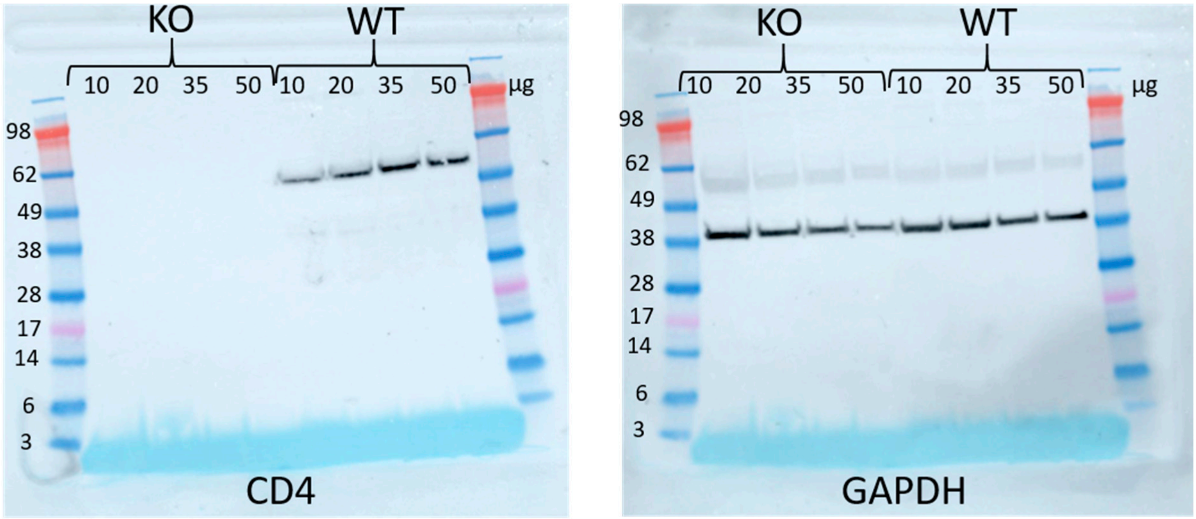

Supplementary Figure S1: Western blot analysis of CD4 KO cells. *Western blot analysis of SUP-T1 WT and KO cell lysates (10, 20, 35, and 50 µg) to detect the presence of CD4 protein (left gel, expected mass of 55 kDa) and GAPDH as loading control (expected mass of 37 kDa).*

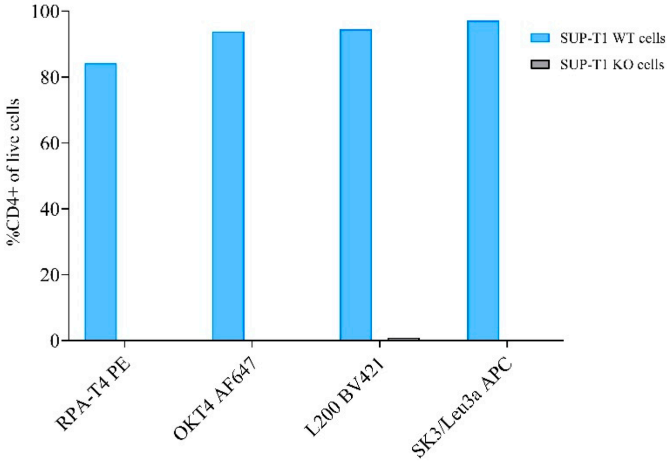

Supplementary Figure S2: Flow cytometric validation of CD4 KO cells. SUP-T1 WT and KO cells were incubated with four different CD4 antibodies (clone OKT4, Cat# 566681; clone RPA-T4, Cat# 561844; L200, Cat#562842; SK3, Cat#565994, BD Biosciences, USA) and analyzed by flow cytometry.

Validation of cell-based assays

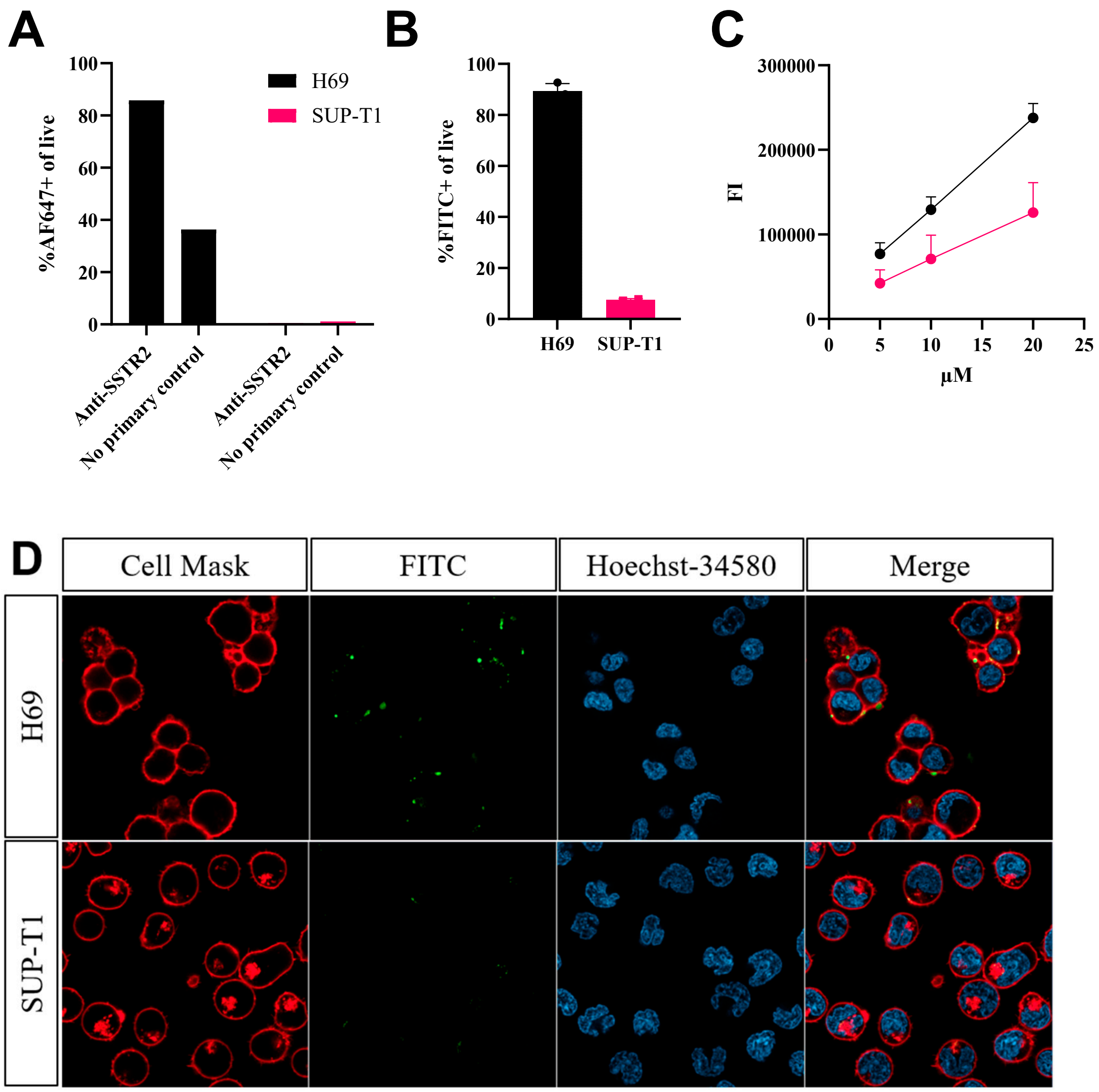

Supplementary Figure S3: Investigation of binding of FITC-Octreotate to H69 and SUP-T1 cells. *In these assays, H69 (with high expression of SSTR2) was used as the target cell and SUP-T1 (with low expression of SSTR2) was used as the control cell. A) H69 and SUP-T1 cells stained with primary anti-SSTR2 antibody (Cat#MAB-4224, R&D Systems) or no primary antibody followed by staining with AF647-anti-mouse IgG (Cat#115-605-164, Jackson). B) FITC-Octreotate binding to H69 and SUP-T1 cells visualized as % FITC positive cells of live. C) Fluorescence intensity measurements in plate assay of cells incubated with FITC-Octreotate (n= 5). D) Confocal microscopy images of cells incubated with FITC-Octreotate (green), Hoechst 34580 (blue = nuclei) and CellMask™ (red=membrane).*

Supplementary files of NGS data: The NGS datasets of the naïve library and the 3rd round eluate are available as CSV files in the Supplementary Materials. These files contain the sequences of peptides, their frequency (copy number), and relative abundance (as percentage).
